# Modelling diagnostics for *Echinococcus granulosus* surveillance in sheep using Latent Class Analysis: Argentina as a case study

**DOI:** 10.1016/j.onehlt.2021.100359

**Published:** 2021-12-04

**Authors:** Abagael L. Sykes, Edmundo Larrieu, Thelma Verónica Poggio, M. Graciela Céspedes, Guillermo B. Mujica, Maria-Gloria Basáñez, Joaquin M. Prada

**Affiliations:** a London Centre for Neglected Tropical Disease Research and MRC Centre for Global Infectious Disease Analysis, School of Public Health, Imperial College London, London, UK; b Facultad de Ciencias Veterinarias, Universidad Nacional de La Pampa, General Pico, Argentina; c Escuela de Veterinaria, Universidad Nacional de Río Negro, Choele Choel, Argentina; d Instituto de Ciencia y Tecnología César Milstein (CONICET), Buenos Aires, Argentina; e Instituto Nacional de Enfermedades Infecciosas, Buenos Aires, Argentina; f Ministerio de Salud, Provincia de Río Negro, Viedma, Argentina; g Faculty of Health and Medical Sciences, University of Surrey, Guildford, UK

**Keywords:** Echinococcosis, Surveillance, Latent class analysis, Bayesian inference, Diagnostics, Argentina, BCI, Bayesian Credible Interval, CE, Cystic Echinococcosis, CI, Confidence Interval, DALY, Disability-adjusted life year, ELISA, Enzyme-Linked Immunosorbent Assay, JAGS, Just Another Gibbs Sampler, LCA, Latent class analysis, MCAR, Missing completely at random, MCMC, Markov Chain Monte Carlo, OD, Optical density, ROC, Receiver Operating Characteristic, SD, Standard deviation, USD, United States Dollar, WB, Western blot, WHO, World Health Organization.

## Abstract

*Echinococcus granulosus* sensu *lato* is a globally prevalent zoonotic parasitic cestode leading to cystic echinococcosis (CE) in both humans and sheep with both medical and financial impacts, whose reduction requires the application of a One Health approach to its control. Regarding the animal health component of this approach, lack of accurate and practical diagnostics in livestock impedes the assessment of disease burden and the implementation and evaluation of control strategies. We use of a Bayesian Latent Class Analysis (LCA) model to estimate ovine CE prevalence in sheep samples from the Río Negro province of Argentina accounting for uncertainty in the diagnostics. We use model outputs to evaluate the performance of a novel recombinant B8/2 antigen B subunit (rEgAgB8/2) indirect enzyme-linked immunosorbent assay (ELISA) for detecting *E. granulosus* in sheep. Necropsy (as a partial gold standard), western blot (WB) and ELISA diagnostic data were collected from 79 sheep within two Río Negro slaughterhouses, and used to estimate individual infection status (assigned as a latent variable within the model). Using the model outputs, the performance of the novel ELISA at both individual and flock levels was evaluated, respectively, using a receiver operating characteristic (ROC) curve, and simulating a range of sample sizes and prevalence levels within hypothetical flocks. The estimated (mean) prevalence of ovine CE was 27.5% (95%Bayesian credible interval (95%BCI): 13.8%–58.9%) within the sample population. At the individual level, the ELISA had a mean sensitivity and specificity of 55% (95%BCI: 46%–68%) and 68% (95%BCI: 63%–92%), respectively, at an optimal optical density (OD) threshold of 0.378. At the flock level, the ELISA had an 80% probability of correctly classifying infection at an optimal cut-off threshold of 0.496. These results suggest that the novel ELISA could play a useful role as a flock-level diagnostic for CE surveillance in the region, supplementing surveillance activities in the human population and thus strengthening a One Health approach. Importantly, selection of ELISA cut-off threshold values must be tailored according to the epidemiological situation.

## Highlights

• Development of a Bayesian LCA model for ovine cystic echinococcosis (CE) in Argentina.
• Evaluation of diagnostic performance for a novel rEgAgB8/2 indirect ELISA.
• Identification of optimal ELISA thresholds for use as a flock-level surveillance tool.
• Improvement of CE surveillance in sheep will help strengthen the One Health approach.

## 1 Introduction

*Echinococcus granulosussensulato* (Cestoda: Taeniidae) is a globally prevalent zoonosis known for causing cystic echinococcosis (CE) in natural (primarily goats and sheep) and accidental (humans) intermediate hosts [[1], [2], [3]]. In CE, hydatid cysts form in the intermediate hosts' organs, leading to clinical manifestations such as discomfort, biliary fistulas and breathing difficulties in humans [[4], [5], [6]]. Intermediate hosts become infected through the ingestion of parasite's eggs shed in the faeces of definitive hosts (canids), which hatch into oncospheres, penetrate the intestinal wall and migrate to various organs (often lungs and liver). In these organs, the oncosphere develops into a thick-walled hydatid cyst that enlarges gradually, producing protoscolices and daughter cysts. If a cyst ruptures, the liberated protoscolices may create secondary cysts in other body sites. Canids become infected upon ingestion of hydatid cysts in infected viscera from intermediate hosts, from which protoscolices evaginate, attach to the small intestinal mucosa and develop into sexually reproducing adults [[7], [8], [9]].

*Echinococcus granulosus* is widely prevalent in pastoral and sheep-rearing areas where domestic dogs and other canid species have frequent access to viscera [3]. Burden of disease estimates in 2013 placed the global medical burden of CE at 800,000 prevalent cases with 2200 annual deaths and 180,000 disability-adjusted life years (DALYs) [10]. The 2017 Global Burden of Disease Study estimated the number of DALYs due to CE at 100,000 (95% uncertainty interval: 72,800–139,000) [11]. Economic modelling has estimated the global annual financial loss due to CE at USD 763,980,979 (95% confidence interval (95%CI): 676,048,731–857,982,275) for human cases alone, with an additional annual livestock production loss of USD 2,190,132,464 (95%CI: 1,572,373,055–2,951,409,989) [12]. In the province of Río Negro, Argentina, the cost of CE was estimated to range between USD 4,234,000 (95%CI: 2,709,000–6,226,000) and USD 5,897,000 (95%CI: 3,452,000– 9,105,000), with livestock losses accounting for up to 94% of this value [13].

Among some of the worst affected countries is Argentina, which has made considerable efforts in the last 50 years to control CE [[14], [15], [16]]. The approach to echinococcosis control in Argentina revolves, firstly, around veterinary activities based on dog deworming and lamb vaccination, and secondly, on the active search for cases in the human population by serology- and/or ultrasonography-based screening supplemented, when appropriate, by opportune surgical intervention or provision of albendazole treatment. This two-pronged line of attack can only be successful with an inter-sectoral and interdisciplinary approach based on the concepts and strategies of “One Health”. In fact, the support and engagement of the community with the control programme is paramount to ensure attendance of sheep farmers with their dogs at designated deworming sites, cooperation for the vaccination of their lambs, provision of consent to serological and/or ultrasound studies in their children, and adjustment of their behaviours with healthy habits in relation to echinococcosis. In Río Negro province a control programme has been in place since 1980. A combination of mass canine administration of praziquantel, ovine vaccination and surveillance, led to a reduction of 90% in dogs and 95% in humans by 1997, and a reduction of 65% in sheep by 2015 [14,17,18]. Despite these measures, CE is still prevalent in the area and across Argentina, with 478 human cases between 2006 and 2016 and 3542 cases between 2009 and 2014 [19,20], highlighting the need for continued CE control.

Effective surveillance is essential for CE control. The current (partial) gold standard for surveillance and diagnosis of ovine CE is necropsy, involving post-mortem visual inspection and palpation of lungs and liver. Often, these organs are sliced into smaller sections for detailed inspection [21]. Necropsy allows for *E. granulosus* to be differentiated from other infections, leading to a high diagnostic specificity [22]. However, in early infections cysts can be small, resulting in decreased sensitivity [23,24]. Additionally, in highly endemic areas there are often no specific slaughter premises or sufficient necropsy expertise, making it difficult to collect such data.

Therefore, less invasive methods, such as serology-based techniques, have been adopted. In sheep, serological diagnostics include the enzyme-linked immunosorbent assay (ELISA) and the western blot (WB). These techniques can be used to detect host antibodies specific to *E. granulosus* in serum samples, allowing for the detection of exposed/infected flocks without the need for necropsy. However, despite improvements to these approaches through the use of recombinant antigens, their diagnostic performance can be highly variable in the field, limiting their effectiveness as surveillance tools [23,[25], [26], [27], [28]].

Due to the lack of highly accurate diagnostics for surveillance in sheep, the true burden of ovine CE remains unknown. This impedes estimation of the magnitude of CE transmission and evaluation of the impact of control measures. Latent class analysis (LCA) models permit assigning ‘true’ infection status to a hidden variable, which can then be estimated within the model. This method was first described in [29] using frequentist statistics but has since been developed to incorporate Bayesian inference [[30], [31], [32], [33]]. While these techniques have previously been applied to diagnostic data from *E. granulosus* and *E. multilocularis* in canids [34,35], they have yet to be applied to livestock diagnostics.

This paper presents a Bayesian LCA model to estimate individual *E. granulosus* status in sheep samples from Río Negro province, Argentina. The model is then used to evaluate the performance of a novel indirect ELISA using a recombinant B8/2 antigen B subunit (rEgAgB8/2). Finally, suitable ELISA cut-offs are identified that may permit the field use of this assay as a surveillance tool for detecting CE both at the individual sheep level and at the flock/farm level according to epidemiological situation.

## 2 Materials and methods

### 2.1 Study sites and data collection

Animals originated from two slaughterhouses in Bariloche and Sierra Colorada (Río Negro, Argentina), where routine surveillance is ongoing. The data comprise 79 adult (aged ≥6 years) unvaccinated sheep from natural vegetation grazing fields. The sheep were analysed *post-mortem* for the presence of *E. granulosus* cysts through necropsy, and for the presence of *E. granulosus* antibodies in serum through a novel indirect ELISA using rEgAgB8/2 (Supplementary File, Text S1) described in [36]. For the WB, total hydatid cyst fluid was used as the antigen preparation and the procedures described in [37] were followed; samples with five precipitation bands in the region of 52–67KDa molecular weight were classified as positive [37]. The necropsy was carried out on site through visual inspection, palpation and slicing of organs. The ELISA and WB were performed blind, without knowledge of the necropsy result. Any missing data or indeterminate results were assumed to be missing completely at random (MCAR). Table S1 provides the results according to the diagnostics used for the 79 sheep examined.

### 2.2 Bayesian Latent Class Analysis

The mathematical framework is a Bayesian latent class model run with a Markov Chain Monte Carlo algorithm following [38]. For each individual sheep, *i*, the hidden variable in the model is the binary ‘true’ infection status, denoted as *status*_*i*_ and defined as follows,

$${\mathrm{status}}_{i}\left\{\begin{array}{c} \;0;\mathrm{uninfected}\\ 1;\mathrm{infected} \end{array}\right)$$

At each iteration of the algorithm, individuals are assigned to the uninfected class or the infected class (status_*i*_ = 0 or 1 respectively). The average value per individual can then be interpreted as the probability of infection for each individual. The estimated ‘true’ prevalence of CE in the data was calculated from the proportion of individuals whose infection status was estimated to be equal to 1 at each iteration.

The ELISA optical density (OD) was assumed to be gamma distributed in each (uninfected, 0, and infected, 1) group, with shape parameters, *sh*_0_ and *sh*_1_, and rate parameters, *rt*_0_, and *rt*_1_, respectively. As a constraint, the mean of the gamma distribution was assumed to be higher in the infected (*mn*_1_) than in the uninfected group (*mn*_0_), such that,

$${\mathrm{ELISA}}_{i}\left\{\begin{array}{c} \mathrm{gamma}\left({\mathrm{sh}}_{0},{\mathrm{rt}}_{0}\right);\mathrm{if}\;\mathrm{status}=0\\ \mathrm{gamma}\left({\mathrm{sh}}_{1},{\mathrm{rt}}_{1}\right);\mathrm{if}\;\mathrm{status}=1 \end{array}\right)$$

To implement the WB diagnostic results (a binary, positive or negative outcome), it was assumed that uninfected individuals have a probability *Q*_0_ of testing positive, while infected individuals have a probability *Q*_1_. The value of *Q*_1_ was assumed always to be higher than that of *Q*_0_, such that infected individuals are more likely to be positive by WB than uninfected individuals. Therefore,

$${\mathrm{WB}}_{i}\left\{\begin{array}{c} \mathrm{Bernoulli}\left(Q_{0}\right);\mathrm{if}\;\mathrm{status}=0\\ \mathrm{Bernoulli}\left(Q_{1}\right);\mathrm{if}\;\mathrm{status}=1 \end{array}\right)$$

To capture the necropsy data in the model, it was assumed that uninfected individuals cannot have cysts (implicitly assuming a specificity of 100%). Infected individuals (*status*_*i*_ = 1) have a probability *Z* of cysts being detected at necropsy, such that,

$${\mathrm{Necropsy}}_{i}\left\{\begin{array}{c} 0;\mathrm{if}\;\mathrm{status}=0\\ \mathrm{Bernoulli}\left(Z\right);\mathrm{if}\;\mathrm{status}=1 \end{array}\right)$$

An alternative model, excluding the WB data, was explored (Text S2).

Two Markov chain Monte Carlo (MCMC) chains were generated using a Gibbs sampler algorithm, which consisted of 100,000 iterations, with a burnin of 10,000 and a thinning of 5. The model was implemented through RStudio version 1.2.5033 [39] and JAGS 4.3.0 [40] using the package “runjags” [41]. Diagnostic plots and the Gelman-Rubin statistic were used to assess convergence. Uninformative priors were used throughout, with the exception of the sensitivity and specificity for the WB and necropsy diagnostics (Table S2).

### 2.3 Determining optimal ELISA cut-off values

#### 2.3.1 Individual-level diagnosis

The indirect ELISA yields an OD value for each individual. Traditionally, a cut-off value is used so that the results can be interpreted, and an individual can be classified as either positive (above the cut-off) or negative (below the cut-off). Here we calculated this threshold using the infection status estimated by the model with a receiver operating characteristic (ROC) curve. We sampled from the joint posterior distribution (*n* = 500) to extract the predicted infection status of each individual sheep. The mean and 95% Bayesian credible intervals (95%BCI) for the sensitivity and specificity of each (posterior) sample *s*, for 79 cut-off thresholds (*k* = 0.199 to 1.176) were calculated. The optimal cut-off threshold was calculated by maximising, across the *s* samples, both the sensitivity (*sens*) and specificity (*spec*), via selecting the lowest value of the following function,

$$\frac{\sum_{s=1}^{s=500}\left({\left(1-{\mathrm{sens}}_{s,k}\right)}^{2}+{\left(1-{\mathrm{spec}}_{s,k}\right)}^{2}\right)}{500}$$

#### 2.3.2 Flock-level diagnosis

While ELISA diagnostics can suffer from poor performance when identifying individual infections, they can provide a useful flock-level tool to help determine which farms are exposed/infected. The optimal cut-off for the use of ELISA at flock level is potentially different from that used in individual diagnosis and depends on underlying prevalence and sample size. We thus simulated 21 farm scenarios with an ovine CE prevalence ranging from 0% to 20% (in 1% increments), which is the current range of prevalence observed in the areas of Río Negro where the data were collected. In these simulated farms, we sampled a number of individual sheep ranging from 1 to 100. Nine possible cut-off points for the ELISA (from 0.24 to 0.603) were selected from the 79 values previously considered, to represent the range of values observed from this assay. Drawing the sensitivity and specificity of each cut-off value from the posterior distribution obtained in the model, we calculated how many animals would test positive. This was undertaken with 100 repeats and we report the proportion of repeats with a correct flock-level diagnosis (i.e. positive if prevalence is above zero, equivalent to the sensitivity of the flock-level diagnostic, or negative if prevalence is equal to zero, namely the specificity). After preliminary analyses, it was assumed that ≥2 positive samples are required for a positive classification at flock level.

## 3 Results

Table 1 summarises descriptive statistics for the diagnostics used. Fig. S1 presents the necropsy results according to parasite species identification.

**Table 1:** Descriptive statistics of the diagnostics used in a sample of 79 sheep from two slaughterhouses in the Río Negro province, Argentina, for detection of *Echinococcus granulosus*.

Result	Necropsy *n* (%)	ELISA optical Density (OD)	Western blot (WB) *n* (%)
Positive	15 (19.0)	NA	19 (24.0)
Negative	48 (60.8)	NA	21 (26.6)
Unknown ^a^	2 (2.5)	NA	39 (49.4)
Other infections ^b^	14 (17.7)	NA	NA
Mean	NA	0.399	NA
Standard deviation	NA	0.168	NA
Range	NA	0.199–1.176	NA

a = missing values (indeterminate results were assumed to be missing); b = other infections detected during necropsy: *Taenia hydatigena* (12/14), *Thysanosoma actinioides* (1/14) and *Fasciola hepatica* (1/14). NA = not applicable.

### 3.1 Latent Class Analysis

Table 2 presents the results of the LCA model. The mean prevalence of *E. granulosus* was estimated at 27.5% (95%BCI: 13.8%–58.9%). The estimated means of the two gamma distributions of the ELISA ODs were 0.373 (95%BCI: 0.310–0.414) for the uninfected group and 0.473 (95%BCI: 0.392–0.591) for the infected group. The estimated sensitivity and specificity values of the WB were similar to one another and around 50%, albeit these values are fairly uncertain due to the limited data (sensitivity = 47.4% (95%BCI: 23.6%–72.1%); specificity = 50.0% (95%BCI: 32.1%–67.4%)). The sensitivity of necropsy was estimated at 79.9% (95%BCI: 39.6%–99.4%) while the specificity was fixed at 100% (and therefore not estimated).

**Table 2:** Outputs of the Latent Class Analysis model summarised as the means and their 95% Bayesian Credible Intervals (95%BCI) obtained from the posterior distributions.

Output	Model estimates (95%BCI)
CE prevalence (%)	27.5 (13.8–58.9)
Mean ELISA OD for uninfected (*mn*_0_)	0.373 (0.310–0.414)
Mean ELISA OD for infected (*mn*_1_)	0.473 (0.392–0.591)
Shape ELISA OD for uninfected (*sh*_0_)	11.117 (6.215–28.706)
Shape ELISA OD for infected (*sh*_1_)	4.876 (2.177–8.605)
WB: Sensitivity (*Q*_1_) (%)	47.4 (23.6–72.1)
WB: Specificity (1 – *Q*_0_) (%)	50.0 (32.1–67.4)
Necropsy: Sensitivity (*Z*) (%)	79.9 (39.6–99.4)

BCI = Bayesian Credible Interval; CE = Cystic echinococcosis; WB = Western blot; ELISA = Enzyme-linked immunosorbent assay; OD = optical density.

The mean probability of being infected ranged, among individual sheep, from 0.044 to 1 (mean ± SD = 0.270 ± 0.364), the value of 1 corresponding to those animals who were positive for necropsy (as its specificity was assumed to be 100%). Fig. S2 and S3 present, respectively, the frequency distribution of these values across individuals using the LCA model and its alternative (excluding WB data).

### 3.2 Performance of the novel indirect ELISA assay

**Fig. 1:**
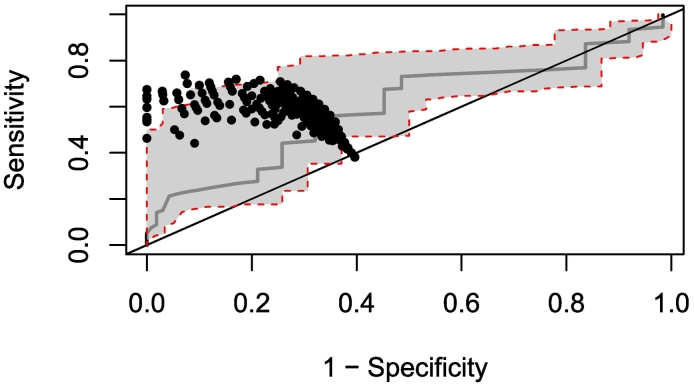
Receiver Operating Characteristic (ROC) curve analysis of ELISA optical density (OD) cut-off thresholds for individual sheep diagnosis. The solid grey line represents, for the 79 thresholds investigated, the mean Sensitivity vs. 1 – Specificity values, and the dashed red lines encompass the 95% quantiles (grey shaded area). The diagonal black line represents random classification. The optimal ELISA threshold was identified as 0.378 (with the black circles indicating the distribution of diagnostic performance). At this cut-off threshold, the average sensitivity was estimated at 55% (95%BCI: 46%–68%) and the average specificity at 67% (95%BCI: 63%–92%). (For interpretation of the references to colour in this figure legend, the reader is referred to the web version of this article.)

The optimal ELISA OD threshold for individual sheep diagnosis was identified as 0.378, which corresponds to a mean sensitivity of 55% (95%BCI: 46%–68%) and a mean specificity of 68% (95%BCI: 63%–92%). The sensitivity and specificity for the range of cut-off thresholds considered is shown as the ROC curve (with 95% quantiles) of Fig. 1.

**Fig. 2:**
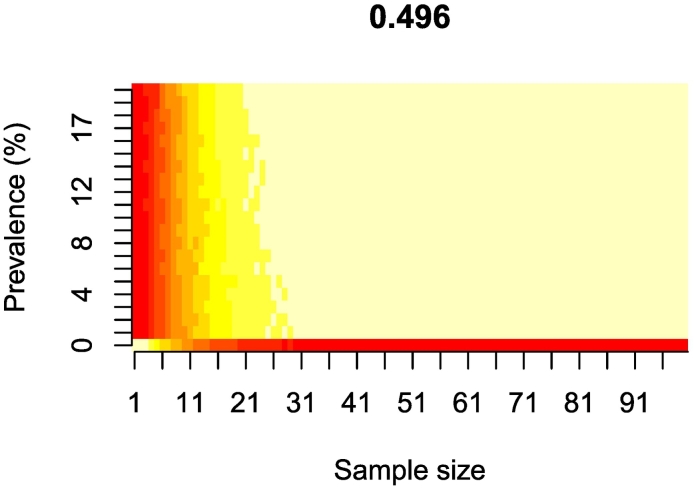
Probability of correctly classifying CE flock status when varying CE prevalence and sample size. Proportion of repeats (out of 100) yielding a correct flock-level diagnosis (i.e. positive if prevalence is above zero, or negative if equal to zero), across simulated sample sizes of 1 to 100 and CE prevalence values of 0% to 20%, for an ELISA optical density (OD) cut-off threshold of 0.496. A positive status at the flock level is indicated by ≥2 positive individual results within the hypothetical flock. The colour scale ranges from dark red (probability of correct classification equal to 0.0) to pale yellow (probability equal to 1.0). (For interpretation of the references to colour in this figure legend, the reader is referred to the web version of this article.)

The sample size simulation results in hypothetical sheep flocks are presented in Fig. S4 as the proportion of repeats with correct flock-level diagnosis (i.e. positive for prevalence values 1% to 20%, and negative for a prevalence value of 0%). Lower cut-off thresholds appear to have a high risk of false positives, especially at low prevalence values. This risk is decreased as the cut-off threshold is increased, with the best performing threshold being identified as 0.496 (Fig. 2). This value suggests that a sample size of approximately 18 sheep would be the most appropriate, with an 80% probability of correctly classifying CE presence in the flock when prevalence ranges from 1 to 20% (sensitivity), but with 20–30% probability of correctly identifying the flock as negative when prevalence = 0% (specificity) (Fig. 2, Table 3). Lower thresholds (panels A–F, Fig. S4) would decrease specificity, and higher thresholds would require greater sample size and reduce the sensitivity (panels G, H) of the diagnostic, limiting its usefulness in low prevalence areas (Fig. S4).

**Table 3:** Sample size estimates and probabilities of correct CE classification when CE is present from the simulations of hypothetical flocks for different values of the ELISA OD cut-off threshold.

Cut-off threshold	Mean optimal sample size ^a^	Proportion of correct disease classification ^b^
0.240	2	0.754
0.301	3	0.734
0.356	3	0.462
0.434	10	0.751
0.450	9	0.664
0.468	10	0.629
0.496	18	0.802
0.545	23	0.714
0.603	38	0.735

CE = Cystic echinococcosis.

a = Mean value of optimal sample sizes (1−100) across prevalence values (1%–20%).

b = Mean probability of correctly classifying a flock as positive across prevalence values 1%–20% (sensitivity) for the mean optimal sample size identified in previous column.

The ELISA OD threshold cut-off value with the greatest probability of correct CE classification is 0.496.

## 4 Discussion

Cystic echinococcosis is an important zoonosis responsible for a substantial public and veterinary health burden [42]. The World Health Organization (WHO) 2021–2030 roadmap on Neglected Tropical Diseases, has proposed that CE should be targeted for intensified control in highly endemic areas, with 17 countries reaching this target by 2030 [43]. To achieve this, it is necessary to clearly identify such areas, as well as implement and evaluate intensified control. Argentina, with its long-standing CE control programme [44] would be a prime candidate for reaching the WHO 2021–2030 CE goal. Notwithstanding uncertainties surrounding the definition of endemicity levels in CE, and what constitutes intensified control, robust diagnostic methods, with good performance, are required to quantify CE burden and measure progress towards its reduction. However, such methods are not yet available, resulting in uncertain estimates of true prevalence and disease burden. Therefore, we conducted a Bayesian LCA to illustrate its application for the estimation of CE prevalence in sheep samples while accounting for diagnostic uncertainty. Sheep are the most important intermediate host of *E. granulosus* in the Río Negro province of Argentina [14,17,18]. Additionally, we used the LCA results to evaluate the performance of a new ELISA diagnostic and guide its potential use as a field surveillance tool.

The LCA model estimated the mean prevalence of CE in the sheep sample as 27.5%. This is higher than was suggested from the necropsy results alone (19%), likely due to the imperfect sensitivity of the latter. Although we cannot claim that our estimate, based on samples from only two slaughter houses, and with a wide 95%BCI (14%–59%), is representative of the sheep population in Río Negro, it is broadly consistent with a decreasing CE prevalence trend. In 1980, the ovine prevalence in the area was 61% [14], and in 2015 it was 43% [18], likely due to the control measures implemented. However, reducing ovine prevalence is notoriously difficult due to ubiquitous egg contamination of pasture [17,18]; in 1997, a prevalence of 18% had been reported in Río Negro when using an ELISA with 63% sensitivity [14].

After obtaining ‘true’ infection estimates for each individual, we were able to evaluate the diagnostic performance of the novel ELISA at the individual sheep and flock levels. At the individual level, sensitivity and specificity were estimated at 55% and 68% respectively (for an OD cut-off value of 0.378). It is well known that the diagnostic performance of *E. granulosus* ELISA assays is highly variable, with previously published sensitivity and specificity values ranging from 41% to 100% [25,26,28]. Variation in individual immune responses, cyst location, and antigen characteristics have been linked to variable ELISA sensitivity and specificity. Additionally, antigenic structure may be similar among other common helminth infections such as *Taenia hydatigena* and *Fasciola hepatica*, leading to cross-reactivity [[45], [46], [47], [48]]. In Argentina, *Ta. hydatigena* has a substantial prevalence in the sheep population [47].

At flock level, surveillance focuses on identifying flocks or sheep farms where there is infection present rather than on diagnosing individuals. It is thus critical to ensure that enough individuals are tested per flock to classify flocks correctly. By simulating a range of infection levels and testing hypothetical flocks using different prevalence levels and sample sizes, we were able to assess the performance of the ELISA in different scenarios. The results suggest that at flock level, higher cut-off thresholds may provide more accurate results. In fact, whereas the optimal cut-off of the ELISA for individual-level diagnosis was 0.378, the value for flock-level diagnosis was 0.496. For the latter, and when sampling around 20 animals, the sensitivity (the probability of correctly classifying a flock as positive when CE is present) was 80%, but the specificity (the probability of correctly classifying a flock as negative when CE is absent) was 20–30%. We chose to emphasize sensitivity at the expense of specificity, as missing an infected flock/sheep farm would have a greater negative impact on the control programme than implementing control measures in an uninfected farm/flock. Higher thresholds would increase the specificity of the diagnostic, which is a priority for high-prevalence areas, but would compromise the sensitivity (not ideal for low-prevalence areas). This highlights the need for considering appropriate cut-off thresholds on a region-by-region basis, in order to tailor the use of the ELISA to particular epidemiological situations (e.g., the use of lower cut-off thresholds in areas of suspected lower prevalence to prioritise sensitivity). Therefore, the use of the novel ELISA for flock-level detection is promising, allowing quick detection of infected flocks through the testing of a relatively small number of animals in an area where flock sizes can be as large as 6000 sheep [49]. Not only will this improve knowledge of CE burden, but it will also allow identification of areas where there is current transmission and/or control programmes may need strengthening, contributing to the implementation of a One Health approach.

During this work we developed a Bayesian LCA model to assess *E. granulosus* in sheep. This is a powerful tool for prevalence estimation which permits accounting for diagnostic uncertainty and affords a more intuitive interpretation of results. Additionally, subjecting all samples to the same diagnostics and blinding the laboratory analysis to the necropsy results allowed for reduction in detection and verification bias [[50], [51], [52]]. The LCA methodology presented here is very flexible. It would certainly be possible to collect additional data using necropsy and immunoassays in other areas of Argentina with suspected low prevalence that may be on track for local elimination, or on the contrary, in areas in which it is anticipated that control interventions may need intensifying. Then a similar analysis could be conducted to find a suitable threshold for flock-level diagnosis that could inform sampling strategies in the region for assessment of ovine CE in surveillance programmes.

### 4.1 Limitations and further research

As with most models, trade-offs between model accuracy and computational simplicity were made by making assumptions that should be revised in future work. For example, we assumed that individual infection status was the only factor influencing the results of the serology-based (ELISA and WB) diagnostics, and that the results of these tests were independent from one another. However, due to the underlying principle of measuring antibody responses to infection for both ELISA and WB [53], any immune variation present in one individual is likely to affect both assays. A better representation of the situation could be achieved by inclusion of a conditional dependency term between the assays, as done in [31,54]. A further limitation was the modest sample size analysed, the small number of diagnostics applied [55], and the issue of missing WB data (as some analyses were not performed due to redirection of laboratory resources to tackle the COVID19 pandemic), which reduced the predictive power of the model regarding the WB assay. Therefore, another area to be addressed in future studies is that of obtaining larger sample sizes with all the tests completed for all the animals examined, with inclusion of necropsy as a (partial) gold standard in as many animals as possible.

## 5 Conclusions

This is the first description of a Bayesian LCA aimed at diagnosing *E. granulosus* in livestock. The model allowed for the estimation of ovine CE prevalence in sheep samples from the Río Negro province, while accounting for uncertainty in diagnostic performance. Additionally, the model permitted assessment of the diagnostic performance of a novel ELISA, which was determined to have potential applications for flock-level detection. The approach presented here can be used to guide future operational research within Río Negro province and other CE-endemic regions globally and contribute towards improving CE surveillance and control, which when combined with appropriate interventions in the human population can strengthen the One Health approach to tackle cystic echinococcosis for the attainment of the WHO 2021–2030 roadmap goals by accelerating programmatic action, intensifying crosscutting approaches, and facilitating endemic country ownership [43].

## Credit author statement

Abagael L. Sykes: Formal Analysis (Modelling), Investigation, Visualisation, Writing- Original Draft, Read and approved the final version of the manuscript; Edmundo Larrieu: Conceptualization, Supervision, Writing- Reviewing and Editing, Read and approved the final version of the manuscript; Thelma Verónica Poggio: Resources, Investigation (Development of the novel ELISA test using rEgAgB8/2), Read and approved the final version of the manuscript; M. Graciela Céspedes: Resources, Investigation (Performance of the laboratory tests), Read and approved the final version of the manuscript; Guillermo B. Mujica: Resources, Investigation (Performance of the necropsies), Read and approved the final version of the manuscript; Maria-Gloria Basáñez: Conceptualization, Supervision, Validation, Project administration, Writing- Reviewing and Editing, Read and approved the final version of the manuscript; Joaquin M. Prada: Conceptualization, Supervision, Methodology, Validation, Writing- Reviewing and Editing, Read and approved the final version of the manuscript.

## Data statement

All the data have been provided in the Main Text and Supplementary File of this article. The model code is publicly available at https://github.com/abby142/E_granulosus_ARG.

## Ethics statement

The animals examined for this work were part of the routine surveillance activities of the CE control programme in Río Negro province, Argentina.

## Funding statement

This work received support from the 10.13039/100000865Bill & Melinda Gates Foundation [grant No. OPP1184344] through the NTD Modelling Consortium (https://www.ntdmodelling.org/) to MGB and JMP. Under the grant conditions of the Foundation, a Creative Commons Attribution 4.0 Generic License has already been assigned to the Author Accepted Manuscript version that might arise from this submission. MGB acknowledges funding from the 10.13039/501100000265Medical Research Council (MRC) Centre for Global Infectious Disease Analysis (MR/R015600/1), jointly funded by the UK MRC and the UK Foreign, Commonwealth & Development Office (FCDO), under the MRC/FCDO Concordat agreement and is also part of the 10.13039/501100001713European and Developing Countries Clinical Trials Partnership (EDCTP2) programme supported by the 10.13039/501100000780European Union. The funders had no role in the study design; collection, analysis and interpretation of the data; writing of the report, or in the decision to submit the article for publication.

## Declaration of Competing Interest

All authors declare no conflicts of interest.

## Appendix A Supplementary data

Supplementary methods, data and results Image 1
