# Supplementary material for: Modelling diagnostics for Echinococcus granulosus surveillance in sheep using Latent Class Analysis: Argentina as a case study
Source: One Health. 2021 Dec 4;14:100359. doi: 10.1016/j.onehlt.2021.100359 (PMC8683760; doi:10.1016/j.onehlt.2021.100359)
Supplement: Supplementary file 1 — Supplementary methods, data and results [file mmc1.docx]

**Appendix A. Supplementary methods, data and results**

**Modelling diagnostics for *Echinococcus granulosus* surveillance in sheep using Latent Class Analysis: Argentina as a case study**

Abagael L. Sykes^a^, Edmundo Larrieu^b, c^, Thelma Verónica Poggio^d^, M. Graciela Céspedes^e^, Guillermo B. Mujica^f^, Maria-Gloria Basáñez^a,^ *, Joaquin M. Prada^g^

^a^ London Centre for Neglected Tropical Disease Research and MRC Centre for Global Infectious Disease Analysis, Imperial College London, London, UK

^b^ Facultad de Ciencias Veterinarias, Universidad Nacional de La Pampa, General Pico, Argentina

^c^ Escuela de Veterinaria, Universidad Nacional de Río Negro, Choele Choel, Argentina

^d^ Instituto de Ciencia y Tecnología César Milstein (CONICET), Buenos Aires, Argentina

^e^ Instituto Nacional de Enfermedades Infecciosas, Buenos Aires, Argentina

^f^ Ministerio de Salud, Provincia de Río Negro, Viedma, Argentina

^g^ Faculty of Health and Medical Sciences, University of Surrey, Guildford, UK

Corresponding author:

* Maria-Gloria Basáńez, Department of Infectious Disease Epidemiology, School of Public Health, Faculty of Medicine (St. Mary’s Campus), Imperial College London, Norfolk Place, London W2 1PG, UK. E-mail: [m.basanez@imperial.ac.uk](mailto:m.basanez@imperial.ac.uk)

**Text S1. Recombinant B8/2 Antigen B subunit indirect ELISA for *Echinococcus granulosus* detection.**

**Gene optimization, cloning and expression.** Sequence data were obtained from NCBI GenBank with accession number AY569349. Gene optimization of the AgB8/2 for heterologous expression in *Escherichia coli* (including sites BamH I and EcoR I) was cloned in pGEX-1λT (Sigma Aldrich) vector and plasmid transformation into BL21 (DE3) cells for expression under the T7 promoter was performed. AgB8/2 was expressed in fusion with N-terminal GST and C-terminal His tag in BL21(DE3) *E. coli* strain by 0.5 mM IPTG (Invitrogen, USA) induction for 4 hours. The produced *E. granulosus* recombinant AgB8/2 antigen (rEgAgB8/2) was solubilized as an inclusion body in 8M Urea, 0.5M ClNa, phosphate-buffered saline (PBS) pH 7.2 and stirred overnight at 4° C. It was subsequently, diluted to 2M Urea 0.5M ClNa, PBS pH 7.2 and centrifuged at 10,000 rpm for 30 minutes. The rAgB8/2 was purified with Protino Ni-TED/IDA (QIAGEN Hilden, Germany) according to the manufacturer’s instructions. Sodium dodecyl sulfate-polyacrylamide gel electrophoresis (SDS-PAGE) and Western Blot (WB) analyses of the recombinant protein were performed, and the recombinant proteins were evaluated using pooled sera of naturally and experimentally *E. granulosus*-infected sheep (positive control), and lambs negative for *E. granulosus* (negative control).

**ELISA using rEgAgB8/2.** The rEgAgB8/2 was kept at -20°C and diluted 1: 400 with coating buffer carbonate/bicarbonate pH 9.0 (Sigma-Aldrich Corp. St. Louis, MO, USA) for coating Nunc-Immuno™ MicroWell™ 96-well solid plates (Sigma-Aldrich) (50 µL/well). The coating buffer was discarded, and plates were washed three times, at 3 minutes per wash, with washing buffer (0.15 M phosphate-buffered saline containing 0.05 % Tween 20 (Sigma)). Plates were blocked with 300 µL/well of blocking solution (900 mL phosphate-buffered saline, 100 mL adult horse serum, 1% phenol red) for 1 hour at room temperature. The blocking buffer was discarded, and the plates were washed three times. Then, 100 µL of control sera, with dilutions ranging from 200 to 25,600 in blocking solution, was added to each well, and plates were incubated at room temperature for 90 minutes. Plates were subsequently washed three times and 100 µL of donkey anti-sheep IgG-horse-radish peroxidase (HRP) conjugate 1  : 3000 (Invitrogen, Carlsbad, CA, USA) in blocking solution was added to each well; plates were left for 1 hour at room temperature. Plates were washed three times and 100 µL of ABTS (2,20-azino-bis(3-ethylbenzothiazoline-6-sulphonic acid), 0.5 mg/mL in 70 mM citrate phosphate buffer, pH 4.2) with 8 µL of 30% hydrogen peroxide per 6 mL of substrate was added to each well immediately prior to use. Plates were incubated in the dark for 20 minutes. When the first hint of colour showed in the negative controls, the reaction was stopped by the addition of 50 µL of 2% sodium fluoride to each well. Plates were then read at 405 nm using an automated ELISA plate reader. The negative control serum from each plate was titrated with the positive control serum on the same plate and provided a visual endpoint for stopping the development of the colour reaction. Standard curves were generated for each ELISA plate using the absorbance values of the positive control, in comparison with the negative control, for dilutions ranging from 1 : 200 to 1 : 25,600. The conditions of acceptance of each plate were as follows: the negative control optical density (OD) should be ≤0.400; the positive control OD should be >0.750 but <1.950; the OD of the positive control should be 3 times higher than the OD of the negative control.

**Table S1.**

Results of the diagnostic tests for the 79 sheep examined.

| **Sheep ID** | **Necropsy** | **ELISA OD** | **WB** |
| --- | --- | --- | --- |
| 01 | negative | 0.282 | negative |
| 02 | negative | 0.308 | positive |
| 03 | negative | 0.21 | negative |
| 04 | negative | 0.668 | negative |
| 05 | negative | 0.366 | positive |
| 06 | negative | 0.371 | positive |
| 07 | negative | 0.264 | positive |
| 08 | *Ta. hydatigena* | 0.298 | negative |
| 09 | negative | 0.393 | negative |
| 10 | negative | 0.412 | negative |
| 11 | *T. actinioides* | 0.443 | positive |
| 12 | *F. hepatica* | 0.479 | negative |
| 13 | *Ta. hydatigena* | 0.318 | negative |
| 14 | *Ta. hydatigena* | 0.301 | negative |
| 15 | negative | 0.3 | positive |
| 16 | negative | 0.333 | negative |
| 17 | *Ta. hydatigena* | 0.532 | negative |
| 18 | negative | 0.359 | positive |
| 19 | *Ta. hydatigena* | 0.31 | negative |
| 20 | negative | 0.308 | positive |
| 21 | negative | 0.46 | positive |
| 22 | negative | 0.245 | positive |
| 23 | *E. granulosus* | 0.208 | negative |
| 24 | negative | 0.213 | negative |
| 25 | *E. granulosus* | 0.381 | positive |
| 26 | *E. granulosus* | 1.176 | negative |
| 27 | negative | 0.228 | negative |
| 28 | indeterminate | 0.45 | positive |
| 29 | negative | 0.363 | positive |
| 30 | *E. granulosus* | 0.434 | negative |
| 31 | negative | 0.468 | negative |
| 32 | negative | 0.199 | positive |
| 33 | negative | 0.308 | positive |
| 34 | *E. granulosus* | 0.269 | negative |
| 35 | indeterminate | 0.306 | positive |
| 36 | *E. granulosus* | 0.268 | positive |
| 37 | *E. granulosus* | 0.668 | negative |
| 38 | *E. granulosus* | 0.388 | negative |
| 39 | negative | 0.332 | positive |
| 40 | *E. granulosus* | 0.746 | positive |
| 41 | *E. granulosus* | 0.343 | NA |
| 42 | negative | 0.374 | NA |
| 43 | negative | 0.237 | NA |
| 44 | negative | 0.27 | NA |
| 45 | negative | 0.289 | NA |
| 46 | negative | 0.358 | NA |
| 47 | negative | 0.349 | NA |
| 48 | *Ta. hydatigena* | 0.24 | NA |
| 49 | negative | 0.272 | NA |
| 50 | *E. granulosus* | 0.231 | NA |
| 51 | negative | 0.603 | NA |
| 52 | negative | 0.406 | NA |
| 53 | negative | 0.54 | NA |
| 54 | *Ta. hydatigena* | 0.496 | NA |
| 55 | negative | 0.631 | NA |
| 56 | negative | 0.545 | NA |
| 57 | negative | 0.418 | NA |
| 58 | *E. granulosus* | 0.433 | NA |
| 59 | negative | 0.289 | NA |
| 60 | negative | 0.225 | NA |
| 61 | *Ta. hydatigena* | 0.349 | NA |
| 62 | negative | 0.284 | NA |
| 63 | negative | 0.365 | NA |
| 64 | *E. granulosus* | 0.356 | NA |
| 65 | negative | 0.538 | NA |
| 66 | negative | 0.545 | NA |
| 67 | negative | 0.559 | NA |
| 68 | *E. granulosus* | 0.356 | NA |
| 69 | negative | 0.257 | NA |
| 70 | negative | 0.316 | NA |
| 71 | *Ta. hydatigena* | 0.338 | NA |
| 72 | negative | 0.337 | NA |
| 73 | *Ta. hydatigena* | 0.378 | NA |
| 74 | negative | 0.89 | NA |
| 75 | *Ta. hydatigena* | 0.276 | NA |
| 76 | *Ta. hydatigena* | 0.547 | NA |
| 77 | negative | 0.677 | NA |
| 78 | *E. granulosus* | 0.464 | NA |
| 79 | negative | 0.762 | NA |

OD = optical density; WB = Western Blot; *E. granulosus* = *Echinococcus granulosus*; *Ta. hydatigena* = *Taenia hydatigena*; *T. actinioides* = *Thysanoma actinioides*; *F. hepatica* = *Fasciola hepatica*; NA = not available.

**Table S2.**

LCA model parameters, assumed prior distributions, and reference sources for the model parameters.

| Parameter | Prior Distribution | Mean | Reference |
| --- | --- | --- | --- |
| CE prevalence | Beta(1, 1) | – | Uninformative prior |
| *mn*_0_ and *mn*_1_ (ELISA)* | Gamma(0.001, 0.001) | – | Uninformative prior |
| *sh*_0_ and *sh*_1_ (ELISA)* | Gamma(0.001, 0.001) | – | Uninformative prior |
| Western blot (WB) sensitivity (*Q*_1_) | Beta(4, 1.5) | 0.71 | Luka *et al.,* 2008 |
| Western blot (WB) specificity (1–*Q*_0_) | Beta(1.5, 3) | 0.65 | Luka *et al.,* 2008 |
| Necropsy sensitivity (*Z*) | Beta(8, 1) | 0.9 | Expert opinion |
| Necropsy specificity | – | 1 | Assumption |

*The rate parameters (*rt*_0_ and *rt*_1_) of the gamma distributions for the ELISA are estimated as the shape parameters (*sh*_0_ and *sh*_1_) divided by the mean parameters (*mn*_0_ and *mn*_1_).

**Fig. S1.** Necropsy results for the 79 sheep examined from Rio Negro Province, Argentina according to parasite identification.


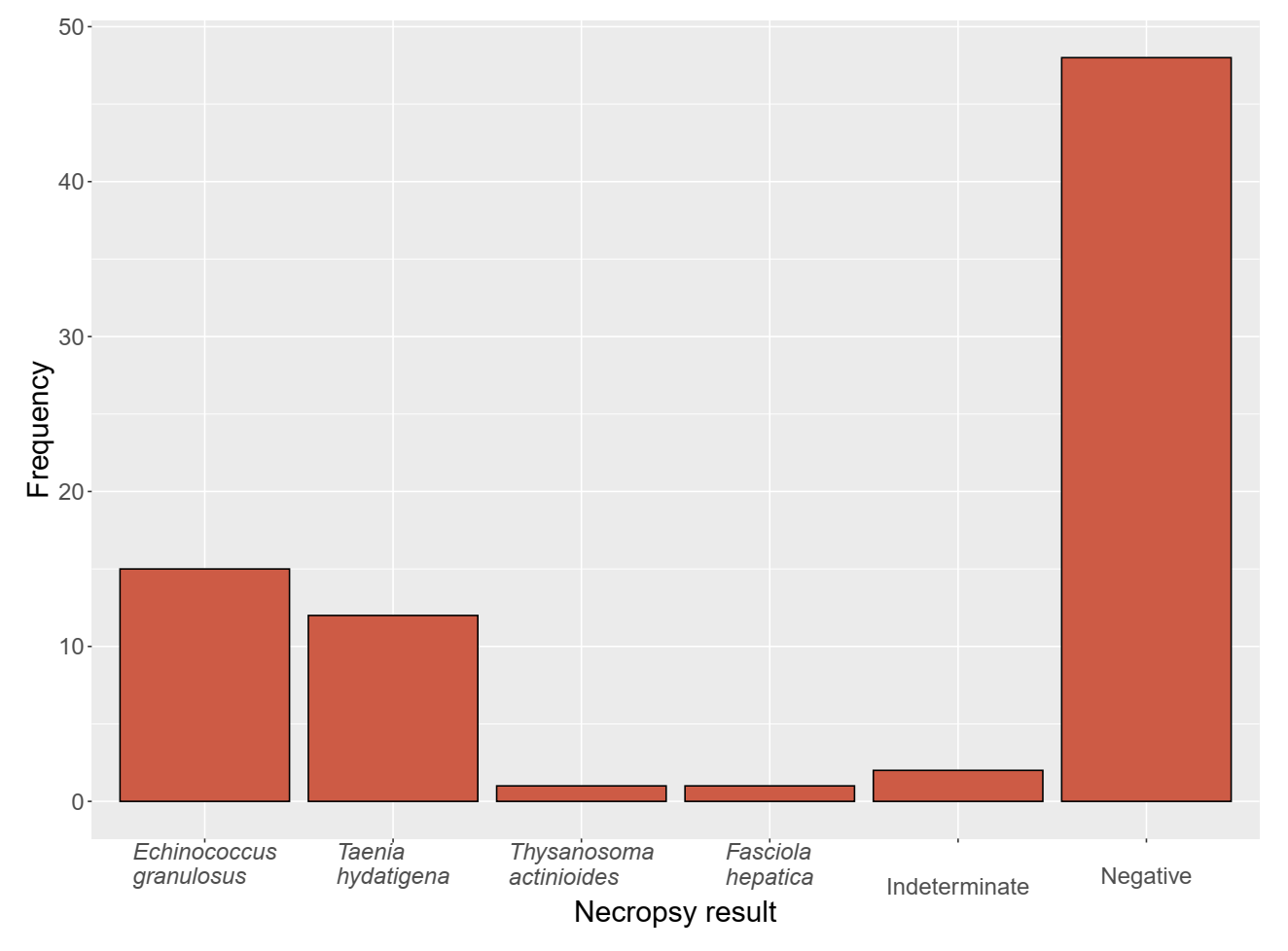


The results were obtained at the Bariloche and Sierra Colorada slaughterhouses through post-mortem visual inspection and palpation of the lungs and liver for detection of infection in sheep (see Main Text). Of the 79 sheep sampled, 28 animals were infected with cestodes (15 with *E. granulosus*, 12 with *Taenia hydatigena*, 1 with *Thysanoma actinioides*); 1 with trematodes (*Fasciola hepatica*); 2 with an indeterminate parasite, and 48 were negative.

**Fig. S2.** Frequency distribution of individual infection status according to the results of the LCA model.


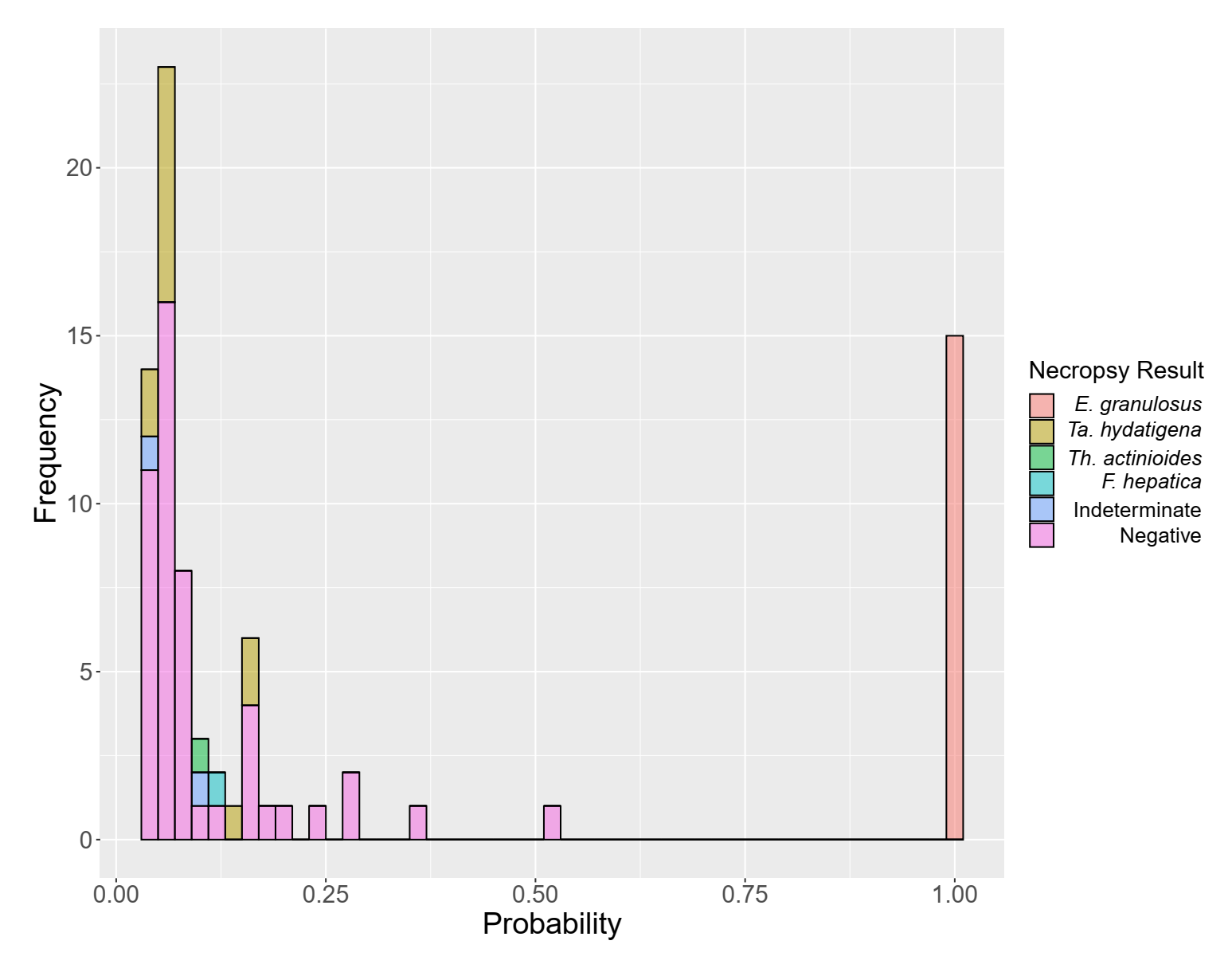


Distribution of individual infection status values as probabilities of being infected according to the outputs of the LCA model described in the Main Text. A sample of 79 sheep from Río Negro province, Argentina was analysed for *E. granulosus* infection by necropsy, the indirect ELISA described in Text S1, and WB (see Table S1 for the results of the diagnostics). Model parameters and prior distributions are listed in Table S2. The predicted mean CE prevalence was 27.5% (95%BCI: 13.8%–58.9%).

**Text S2. Alternative LCA model.**

Since a substantial proportion of the WB data was missing (see Table S1), the model was run including only necropsy and ELISA data to assess the added value of the WB data to model outputs. The WB analyses were not completed due to the redirection of laboratory resources to test for SARS-CoV-2 during the COVID-19 pandemic in 2020. The resulting frequency distribution of posterior probabilities in presented in Figure S3.

**Fig. S3.** Frequency distribution of individual infection status according to the LCA alternative model (including only necropsy and ELISA results).


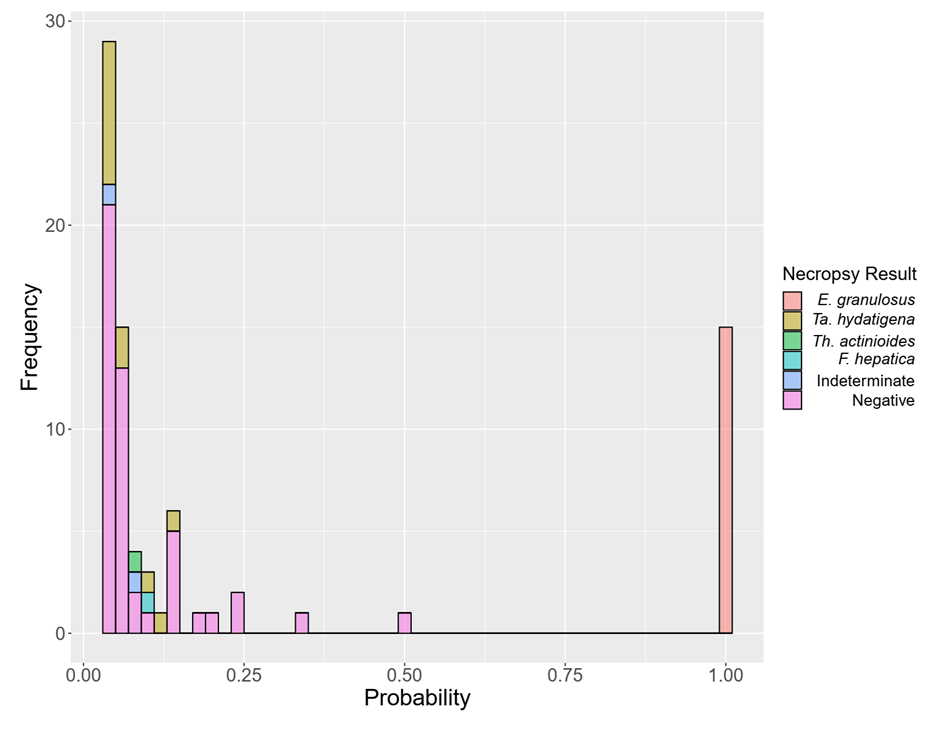


Distribution of individual infection status as probabilities of being infected according to the LCA model including only the necropsy and ELISA data. The predicted mean CE prevalence was 26.4 (95%BCI 13.7–54.9), very similar to that presented in Fig. S2 and Table 2 of the Main Text, indicating that exclusion of the WB data did not have a substantial influence on model outputs. As in Figure S2, 15 animals are clearly identified as being infected with *E. granulosus.*

**Fig. S4.** Probabilities of correctly classifying hypothetical sheep flocks in simulations varying the novel ELISA OD cut-off threshold (0.24–0.60), the prevalence of infection in the flock (0–20%), and the sample size (1–100).


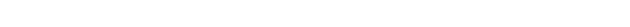

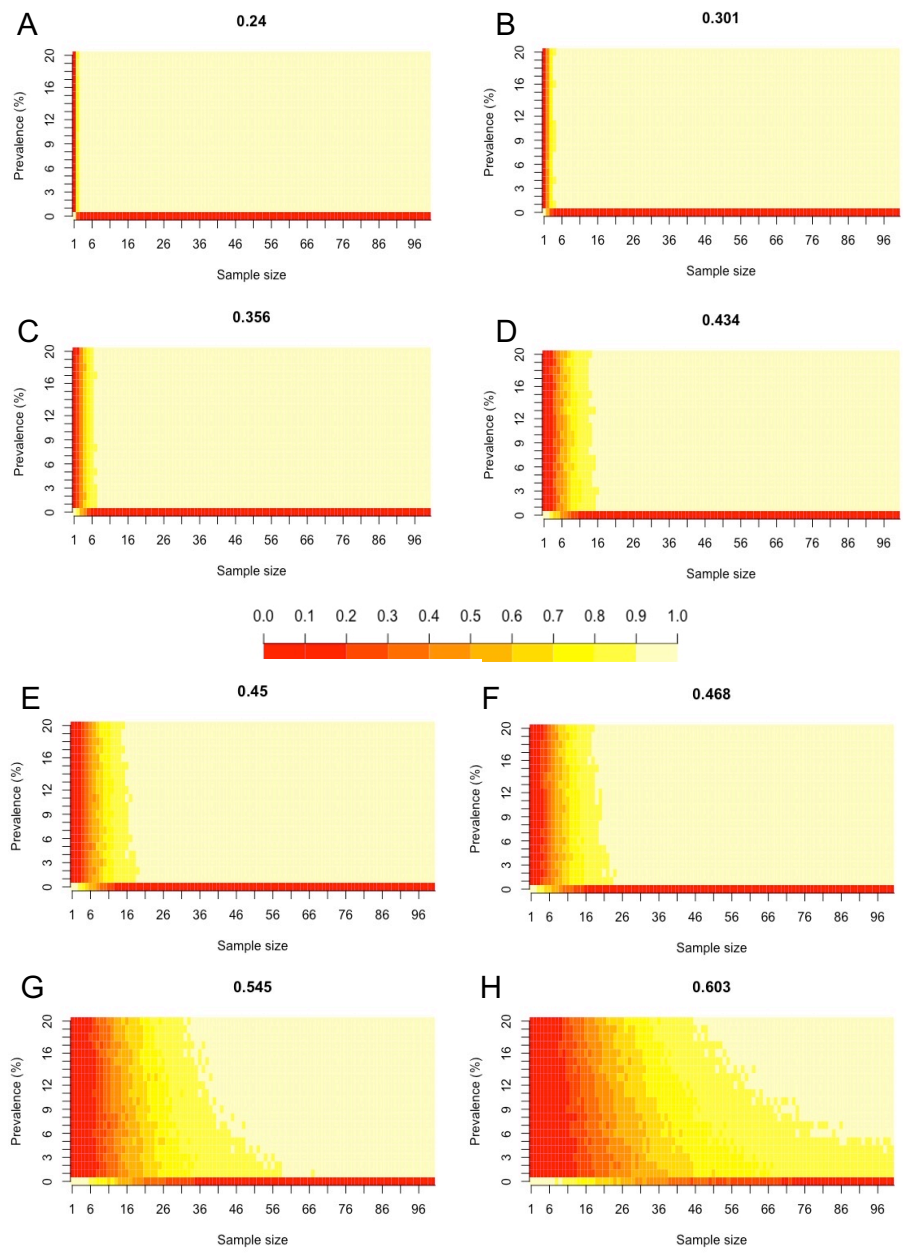


The proportion of repeats (from a total of 100) with correct flock-level diagnosis (i.e. positive if prevalence is above zero, or negative if equal to zero). The sample size (1 to 100) is represented in the horizontal axis of each panel; the simulated CE prevalence in the flock (0% to 20%) corresponds to the vertical axis. Each panel depicts a different ELISA optical density (OD) cut-off threshold for a total of 8 panels selected from the 21 cut-offs investigated (see Main Text). A positive status is determined by ≥2 positive individual results within the flock. The colour scale ranges from dark red (probability of correct classification equal to 0.0) to pale yellow (probability equal to 1.0). The probability of correctly classifying a flock as CE-positive when CE is present (prevalence 1–20%) corresponds to the sensitivity of the cut-off ELISA threshold for flock-level diagnosis; the probability of correctly classifying a flock as CE-negative when CE prevalence is 0% (bottom bar in each panel) is the associated specificity. Panels A–F illustrate simulation results with ELISA cut-off values lower than the optimal of 0.496 presented in Fig. 2 of Main Text, and panels G and H illustrate the results for higher cut-off values (see also Table 3 of Main Text).

**Supplementary references**

[S1] S. Luka, I. Ajogi, I. Nock, C. Kudi, J. Umoh, Evaluation of enzyme-linked immunosorbent assay (ELISA) and Western blotting for the immunodiagnosis of hydatid diseases in sheep and goats, Internet J. Vet. Med. 5 (2008) 1–7. <https://ispub.com/IJVM/5/2/5183> (accessed 15 July 2021).
